# Acute interstitial nephritis associated with ingestion of *Achyranthes japonica* extract: a case report

**DOI:** 10.1186/s12882-021-02326-w

**Published:** 2021-04-07

**Authors:** Ha Nee Jang, Sehyun Jung, Seunghye Lee, Se-Ho Chang, Tae Won Lee, Eunjin Bae, Dong Jun Park

**Affiliations:** 1 Department of Internal Medicine, Gyeongsang National University Hospital, Jinju, South Korea; 2 Department of Internal Medicine, College of Medicine, Gyeongsang National University, Jinju, South Korea; 3 Institute of Health Science, Gyeongsang National University, Jinju, South Korea; 4 Department of Internal Medicine, Gyeongsang National University Changwon Hospital, 11 Samjungja-ro Sungsan-gu, Changwon, 51472 South Korea

**Keywords:** *Achyranthes japonica*, Nephritis, Drugs, Nutraceuticals, Side effects

## Abstract

**Background:**

The Japanese chaff flower, *Achyranthes japonica*, is used as complementary medicine to control degenerative arthritis. Although commonly used in South Korea, there has been no report of side effects. We report the first case of acute interstitial nephritis (AIN) that occurred in a woman who ingested *A. japonica* extract for 4 months.

**Case presentation:**

A 56-year-old Korean woman was admitted for deterioration of renal function. She had general weakness and nausea for 1 month. Her initial blood urea nitrogen and serum creatinine levels were 26.3 mg/dL and 3.2 mg/dL, respectively. She acknowledged ingesting *A. japonica* extract for the past 4 months. Renal histology demonstrated AIN represented by immune cell infiltration into the interstitium, tubulitis, and tubular atrophy, but the glomeruli were intact. *A. japonica* was discontinued immediately and conservative management was started. Renal function was nearly restored to the baseline level without medication after 13 months.

**Conclusion:**

This is a rare case report of AIN associated with a pure *A. japonica* extract. In the case of unknown etiology of AIN, physicians should ask about the use of herbal medicines, nutraceuticals, and traditional folk medicines including *A. japonica*.

## Background

Acute interstitial nephritis (AIN) occurs when acute kidney injury (AKI) is accompanied by histological findings of interstitial inflammation, edema, and tubulitis. AIN is a common cause of AKI [1, 2]. Drug-induced AIN is known to account for 60–70% of cases [1], but the incidence of AIN caused by herbs or folk medicines is unknown and often ignored. Single or concurrent use of herbs with therapeutic drugs could increase the potential for side effects. Traditional herbal medicines have been widely used in South Korea for a long time. The Japanese chaff flower, *Achyranthes japonica*, has been used as complementary medicine for edema and arthritis and to delay a woman’s menstruation without clinical evidence. Recent studies have reported the anti-inflammatory, pain relief, and antibacterial effects of *A. japonica* as well as its ability to improve osteoporosis conditions in ovariectomized rats [3–6]. However, there has been no report on side effects. Therefore, we report a case of AIN after ingesting *A. japonica*. This case report suggests that ingesting a complementary medicine could be a cause of AKI leading to chronic kidney disease (CKD) if management is delayed.

## Case presentation

A 56-year-old Korean woman was admitted for deterioration of renal function. She had suffered from general weakness and nausea for the past month and visited a local clinic. She was recommended to visit the tertiary hospital because of poor renal function. She was a farmer, but she strongly denied any recent exposure to pesticides. She had intermittently taken medicines for arthralgia in both knees. She had been diagnosed with hypertension 3 years previously and was taking 5 mg lercanidipine without change. She had a routine check-up 1 year ago and did not show any abnormalities of renal function at that time. She also denied various infections and other systemic or auto-immune diseases that cause acute TIN by a thorough history taking. However, she complained of a 5-kg weight loss in the last 3 months. She did not complain of fever, oliguria, skin rash, or a urine color change at admission. She had not taken NSAIDs, toxins, or Chinese herbal medicines, but had ingested the extract of *A. japonica* for control of knee pain beginning 4 months ago.

Her initial vital signs were as follow: blood pressure of 140/80 mmHg, heart rate of 68 beats/min, respiratory rate of 20 breaths/min, and body temperature of 36.1 °C. No remarkable findings were observed in the physical examinations. Her complete blood count (CBC), biochemical findings, and arterial blood gas analysis was shown Table 1. Briefly, her blood urea nitrogen and creatinine were elevated whereas potassium level decreased. There was no eosinophilia on her CBC. A arterial blood gas analysis was compatible with metabolic acidosis and anion gap was normal. C3 and C4 levels were 102 mg/dL (normal, 90–180 mg/dL) and 26.9 mg/dL (normal, 10–40 mg/dL), respectively. Immunoglobulin G (IgG), IgA, and IgM levels were 2530 mg/dL (normal, 700–1600 mg/dL), 689 mg/dL (normal, 70–400 mg/dL), and 171 mg/dL (normal, 40–230 mg/dL. Anti-nuclear antibody, anti-neutrophilic cytoplasmic antibody, and anti-glomerular basement membrane antibody were all negative. A urinalysis revealed 1+ protein, 2+ blood, and 3+ glucose by dipstick. Microscopy revealed 10–29 red blood cells/high power field (HPF) and many WBCs/HPF. No bacteria grew on a urine culture. No eosinophils existed on Hansel staining of the urine.

**Table 1:** Serum laboratory findings

**Complete blood count (CBC)**	
WBC (4.0–10.0 × 10^9^/L)	4.94 × 10^9^/L
Neutrophil (50–75%)	49.2%
Lymphocyte (20–44%)	37.7%
Monocyte (2–9%)	6.5%
Eosinophil (1–5%)	3.8%
Hemoglobin (12–16 g/dL)	^a^9.1 g/dL
Hematocrit (36–48%)	^a^26%
Platelet (130–400 × 10^9^/L)	142 × 10^9^/L
**Biochemical findings**	
BUN (8.0–20.0 mg/dL)	^a^26.3 mg/dL
Creatinine (0.51–0.95 mg/dL)	^a^3.2 mg/dL
Sodium (135–145 mmol/L)	137 mmol/L
Potassium (3.3–5.1 mmol/L)	^a^3.0 mmol/L
Chloride (98–110 mmol/L)	112 mmol/L
Total CO_2_ (21–31 mmol/L)	^a^17 mmol/L
Glucose (70–110 mg/dL)	190 mg/dL
Protein (6.6–8.7 g/dL)	8.1 g/dL
Albumin (3.5–5.2 g/dL)	3.9 g/dL
Calcium (8.6–10.2 mg/dL)	9.5 mg/dL
Phosphorus (2.7–4.5 mg/dL)	2.4 mg/dL
HbA1c (4.2–5.9%)	5.0%
**Arterial blood gas analysis**	
pH (7.35–7.45)	^a^7.29
Bicarbonate (24–30 mmol/L)	^a^17 mmol/L
pCO2 (32–46 mmHg)	^a^35 mmHg

*WBC* White blood cell, *BUN* Blood urea nitrogen. ^a^abnormal value

Protein (1406 mg) was detected in the 24-h urine sample. There was no evidence of monoclonal gammopathy in the serum or urine protein on the immunoelectrophoresis assay.

**Fig. 1:**
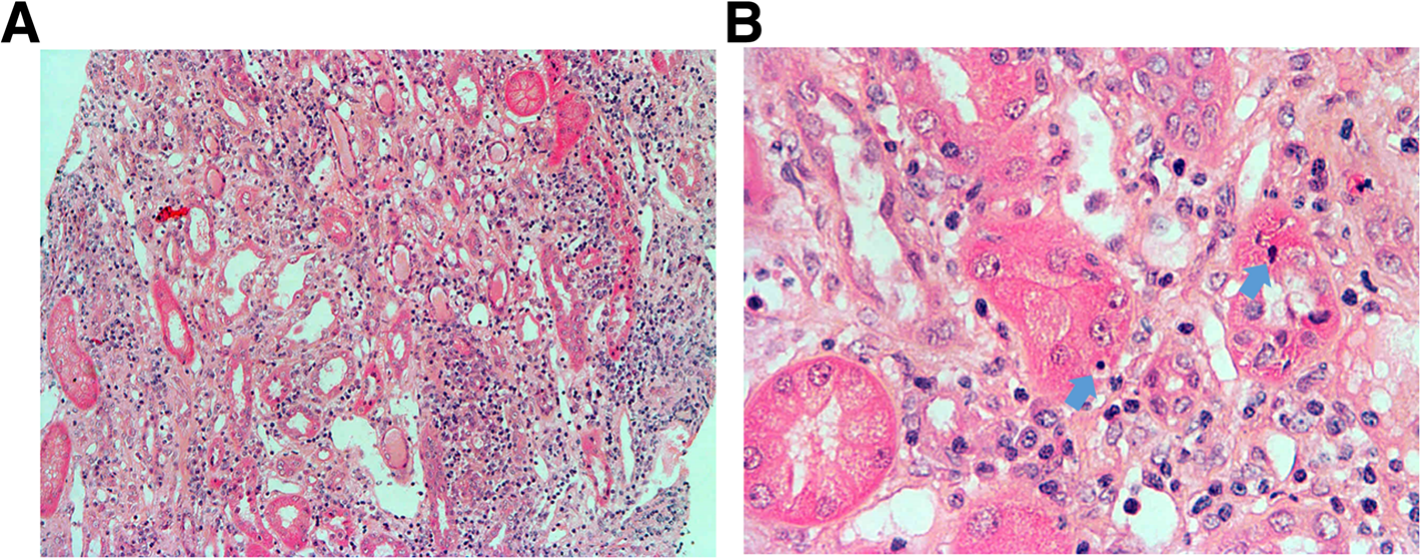
Histologic findings of renal biopsy. There is moderate and diffuse lymphocytic infiltration into interstitium with mild tubular atrophy (**a**) (× 100, H&E staining). Lymphocytic tubulitis (bold arrow) are frequently found on higher magnification (**b**) (× 400, H&E staining)

Kidney size was normal on ultrasonography (USG) (10.5 cm right and 11.0 cm left) with normal echogenicity, and a Doppler study was unremarkable. USG-guided renal biopsy revealed moderate and diffuse interstitial lymphocyte infiltration with mild tubular atrophy, and lymphocytic tubulitis was also frequently found on higher magnification (Fig. 1a and b). The glomeruli were normally sized and cellularity and capillary walls were not thickened. The blood vessels were unremarkable. Imminofluorescence microscopy was negative. As she had not taken any medicines except for anti-hypertensive drugs for the past 3 years without changes, *A. japonica* was considered the causative agent of the tubulointerstitial nephritis (TIN) and was immediately withdrawn. Conservative management, including appropriate blood pressure control, a low salt diet, and oral potassium and bicarbonate replacement was started. Her initial symptoms and signs improved and blood pressure was well controlled without further medication.

**Fig. 2:**
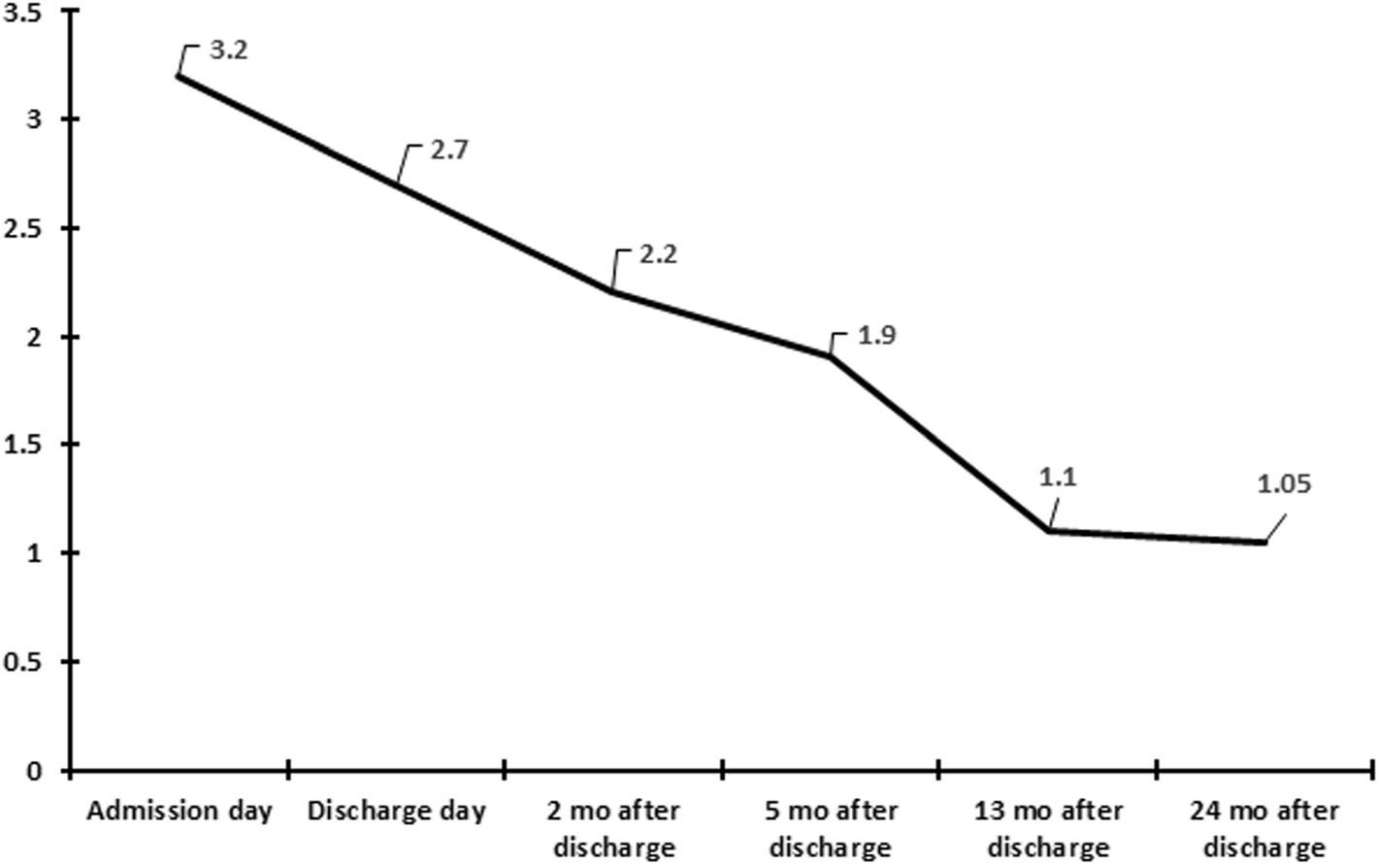
Serial changes of serum creatinine during admission and after discharge

She was discharged on day 10 after admission and followed up in the outpatient department. The abnormal urinalysis findings resolved completely, and oral potassium and bicarbonate administration was withdrawn 13-months after discharge. Serum creatinine was 1.05 mg/dL and the estimated glomerular filtration rate was 57 ml/min/1.73 m^2^ 24-months after discharge (Fig. 2). She is currently being followed up in our outpatient department with the maintenance of renal function.

## Discussion and conclusions

We described a case of AIN that occurred after ingesting *A. japonica* for 4 months. As far as we know, this is the first report in the English literature. There are numerous cases of unexplained decreased renal function in a clinical setting. This report is significant in that careful and thorough history taking of medications, including Chinese herbal medicines, nutraceuticals, and traditional folk medicines are essential to diagnose and treat AIN.

AIN is usually confirmed when AKI is accompanied by histological findings of interstitial inflammation, edema, and tubulitis. Many etiologies of AIN have been recognized, including drugs, various infections, autoimmune or systemic diseases, and idiopathic diseases [1, 2]. A report of pooled data from three large studies revealed that drugs were the most common etiology of AIN, underlying 91 of 128 cases (71.1%) [7]. AIN induced by drugs has been observed in 6.5–27% of patients when a renal biopsy was performed due to unexplained AKI [8–10]. AIN has been reported after ingesting herbal and folk medicines [11–13]. *A. japonica* has been ingested in South Korea for a long time to control arthralgia. However, there have been no reports of adverse effects.

Although acute TIN has a good prognosis when diagnosed early, the causative agents are withdrawn and, where necessary, early steroid therapy is started; undetected subclinical acute TIN can progress to renal fibrosis and eventually cause irreversible CKD [2]. Schwarz et al. revealed that drug-related AIN causes permanent renal insufficiency in 36% of cases with a maximum of 56% in NSAID-induced cases and taking the suspected drug for more than 1 month before the diagnosis causes permanent renal insufficiency in 88% of cases. Less oliguria and anuria as acute symptoms and prolonged intake of the suspected drug are related to a more chronic course of interstitial nephritis [14]. Other studies also showed that acute TIN-related damage is a potential promoter of CKD and the duration of treatment and the cumulative dose appear to increase the risk of kidney damage [15, 16]. Our patient had ingested *A. japonica* for 4 months and the renal histology revealed moderate interstitial cell infiltration and mild tubular atrophy, resulting in long-term restoration of renal function and some irreversibility. Two formal methods are available for calculating the probability of causation: the Naranjo probability scale [17] and the World Health Organization-Uppsala Monitoring Center (WHO-UMC) causality categories [18]. The patient’s Naranjo probability scale score was 6 (probable causal relationship) and her WHO-UMC causality category was “probable”.

AIN is also characterized by tubulitis, which is the extension of interstitial inflammation over the tubular basement membranes. Patients with AIN can present with tubular dysfunction, which includes salt-wasting nephropathy, abnormal renal acidification, urinary concentration defects, potassium secretory defects related to distal nephron injury, or defective proximal tubular reabsorption leading to features of Fanconi’s syndrome presented as glycosuria, phosphaturia, aminoaciduria, hypokalemia, and type II renal tubular acidosis from bicarbonaturia [19]. Our patient also presented with both tubulitis and laboratory features of tubular dysfunction, such as hypokalemia, glycosuria, non-nephrotic proteinuria, and normal anion gap metabolic acidosis. The tubular dysfunction improved 13 months after withdrawing the *A. japonica* and restoration of renal function.

The pathogenesis of drug-induced AIN generally involves allergic reaction that is prompted by exposure to certain drug. As a result, T-cell mediated hypersensitivity reactions and cytotoxic T-cell injuries are involved in pathogenesis of drug-induced AIN [1, 19, 20]. The precise disease mechanism to give rise to AIN by *Achyranthes japonica* is not clear based on our one reported case, but antigen-driven immunopathology by exposure to this drug seems to be NSAID-associated AIN in that there was no clinical symptoms and signs such as fever, rash, arthralgia, and eosinophilia, it took about 6–18 months to develop AIN from the exposure than other drugs, and eosinophil rarely infiltrated into renal interstitium [21]. These clinical and pathological characteristics might be derived from anti-inflammatory properties of this agent.

We firstly report the case of AIN associated with a pure *A. japonica* extract which is widely taken as complementary medicine in South Korea. AIN should be considered in the differential diagnosis of all cases of AKI. Physicians should not only ask about prescribed and an over-the-counter drugs that cause AIN, but also about herbal medicines, nutraceuticals, and traditional folk medicines including *A. japonica*.

## Data Availability

The data supporting the conclusions of this article is included within the article.

## Abbreviations

AIN: Acute interstitial nephritis
AKI: Acute kidney injury
CKD: Chronic kidney disease
HPF: High-power field
IgG: Immunoglobulin G
NSAIDs: Non-steroidal anti-inflammatory drugs
TIN: Tubulointerstitial nephritis
USG: Ultrasonography
WBC: White blood cell
WHO-UMC: World Health Organization-Uppsala Monitoring centre
